# Dot/Icm-Dependent Restriction of Legionella pneumophila within Neutrophils

**DOI:** 10.1128/mBio.01008-21

**Published:** 2021-05-26

**Authors:** Christopher T. D. Price, Hannah E. Hanford, Aruna Vashishta, Mateja Ozanic, Marina Santic, Silvia Uriarte, Yousef Abu Kwaik

**Affiliations:** a Department of Microbiology and Immunology, University of Louisville, Louisville, Kentucky, USA; b Department of Medicine, University of Louisville, Louisville, Kentucky, USA; c University of Rijeka, Rijeka, Croatia; d Center for Predictive Medicine, College of Medicine, University of Louisville, Louisville, Kentucky, USA; e University of Rijeka, Faculty of Medicine, Department of Microbiology and Parasitology, Rijeka, Croatia; University of Illinois at Chicago

**Keywords:** reactive oxygen species, granules, phagosome, polymorphonuclear leukocytes

## Abstract

The Dot/Icm type IV secretion system (T4SS) of Legionella pneumophila is essential for lysosomal evasion and permissiveness of macrophages for intracellular proliferation of the pathogen. In contrast, we show that polymorphonuclear cells (PMNs) respond to a functional Dot/Icm system through rapid restriction of L. pneumophila. Specifically, we show that the L. pneumophila T4SS-injected amylase (LamA) effector catalyzes rapid glycogen degradation in the PMNs cytosol, leading to cytosolic hyperglucose. Neutrophils respond through immunometabolic reprogramming that includes upregulated aerobic glycolysis. The PMNs become activated with spatial generation of intracellular reactive oxygen species within the *Legionella*-containing phagosome (LCP) and fusion of specific and azurophilic granules to the LCP, leading to rapid restriction of L. pneumophila. We conclude that in contrast to macrophages, PMNs respond to a functional Dot/Icm system, and specifically to the effect of the injected amylase effector, through rapid engagement of major microbicidal processes and rapid restriction of the pathogen.

## INTRODUCTION

Legionella pneumophila is an aquatic organism that has evolved to proliferate within a wide range of amoebae species as its primary hosts (1–4). While amoebae and other protists are the natural hosts for L. pneumophila, humans can become an accidental host through inhalation of contaminated environmental aerosols (5, 6). Upon inhalation, the organism invades alveolar macrophages via coiling phagocytosis (7, 8) and begins to proliferate, resulting in pneumonia designated Legionnaires’ disease (9–11). The Dot/Icm type IV translocation system of L. pneumophila, which functions as a molecular syringe, injects ∼350 different protein effectors into the host cell cytosol to evade lysosomal fusion and overrides restriction of pathogen proliferation in both amoebae and alveolar macrophages (12–19).

Various intracellular pathogens have evolved to evade the innate immune response of macrophages (20–22) where they manipulate processes such as vesicle traffic, the lysosomal degradation pathway (23, 24), and the innate immune pathogen sensing mechanisms (25–28). However, neutrophils or polymorphonuclear leukocytes (PMNs) are largely restrictive to most pathogens with very few exceptions. In addition to alveolar macrophages, PMNs are present in the alveoli of pulmonary biopsy specimens from patients and experimental animals infected with L. pneumophila. Since 1981, PMNs have been reported to restrict L. pneumophila, and several studies *in vivo* in mice have shown that PMNs are required for the rapid innate defense in controlling the L. pneumophila infection (12, 14, 16–18, 29–35). Almost 4 decades later, the mechanisms of pathogen uptake and restriction within PMNs remain unknown. PMNs account for 50 to 60% of peripheral blood leukocytes in humans and are an essential part of the innate immune defense against microbial infections.

Upon infection of macrophages and amoeba hosts by L. pneumophila, the Dot/Icm-injected repertoire of effectors is involved in manipulation of various host cell processes to remodel the macrophage into a proliferation niche (6, 36–41). Although L. pneumophila injects Dot/Icm effectors into the cytosol of PMNs (17), proliferation of the pathogen is restricted in PMNs (12–19, 42). Thus, the ability of PMNs to restrict L. pneumophila proliferation is very different from the permissiveness of macrophages. In mice, infiltrating PMNs in the mouse lungs respond to the effect of Dot/Icm translocation of effectors through the secretion of many proinflammatory cytokines, including interleukin 1 alpha (IL-1α), tumor necrosis factor alpha (TNF-α), IL-12, and IL-17 (12, 14, 16–18). In addition, the release of PMNs proinflammatory cytokines has a paracrine effect on PMNs and macrophages that amplify the inflammatory response and enhance pathogen restriction and clearance (43, 44).

Neutrophil extracellular traps (NETs) have been thought to contribute to pathogen-killing mechanisms by PMNs (45–47). However, the two major antimicrobial arms of PMNs are the generation of reactive oxygen species (ROS) by the phagocyte NADPH oxidase and the antimicrobial matrix contents of the specific and azurophilic granules (44). In quiescent PMNs, NADPH oxidase is kept inactive through compartmentalization of three membrane subunits (gp91phox/NOX2, p22phox, and Rap1A) and four cytosolic proteins (p47phox, p67phox, p40phox, and Rac2). Upon stimulation, the cytosolic subunits translocate to the membrane to form the catalytically active enzyme complex, which generates the ROS (48–54). The specific granules contain several antimicrobial proteins like cathelicidin, lactoferrin, and lysozyme, while the azurophilic granules contain the potent cationic antimicrobial peptides α-defensins, myeloperoxidase, proteinase 3, and elastase, among others (43, 44). Although restriction of L. pneumophila was reported since 1981, the mechanism remained unknown (55). It is not known whether any of the two major PMNs’ antimicrobial machineries are involved in restricting L. pneumophila growth within PMNs.

Macrophages respond to infection by various pathogens through immunometabolic reprogramming (56–60), which also occurs in tumors (61). Hyperglycemia in diabetic patients triggers activation of PMNs, similar to *ex vivo* exposure of quiescent PMNs to a high level of glucose (62–64), and this response is mediated through upregulation of aerobic glycolysis (62–65). Similar to the response of macrophages to high levels of glucose through upregulation of aerobic glycolysis, accumulation of lactate, and diminished tricarboxylic acid (TCA) cycle (66–79), PMNs also exhibit upregulation of aerobic glycolysis upon their activation (62–64), and inhibition of glycolysis blocks PMN activation (62). Recent studies have shown that the key glycolytic enzyme 6-phosphofructo-2-kinase (PFK-2) of PMNs interacts with the phagocyte NADPH oxidase (80). Inhibition of PFK-2 reduces NADPH oxidase activity along with reduced glycolysis, and interference with the NADPH oxidase activity diminishes glycolysis (80). Therefore, there is a unique positive feedback loop between the NADPH oxidase activity and upregulation of PMNs glycolysis and their activation (80). Interestingly, diabetic patients are more prone to L. pneumophila infections (34, 35, 81–83), which may be due to the dysregulated inflammatory response of macrophages and PMNs to persistent high levels of glucose (66–79, 84–86).

Degradation of glycogen/glycogenolysis in mammals is a tightly regulated process to avoid the sudden release of a large amount of intracellular glucose (87). We have recently shown that L. pneumophila injects into the cytoplasm of macrophages and the amoeba natural host an amylase (LamA) effector, which rapidly catalyzes rapid unregulated degradation of host glycogen (88). In the natural amoeba host, LamA-mediated glycogenolysis interferes with encystation of amoeba by depriving the natural host of the glucose resource to synthesize the cellulose-rich cyst wall. This renders the amoeba host to remain in the permissive trophozoite form and unable to differentiate into the nonpermissive cyst form (88). In human macrophages, LamA-mediated glycogenolysis generates cytosolic hyperglucose that enhances glycolysis. The macrophages respond by undergoing a paradoxical M1-like proinflammatory response but remain permissive for intracellular survival and replication of L. pneumophila (88).

Here, we show that upon injection of LamA into the cytosol of PMNs, the enzyme rapidly degrades the PMNs glycogen, resulting in a cytosolic hyperglucose. The PMNs respond by enhanced glycolysis, which leads to engagement of the two major PMNs bactericidal mechanisms, the robust spatial generation of ROS and fusion of the pathogen-containing phagosomes (LCP) to the specific and azurophilic granules, which collectively restrict the pathogen. Thus, PMNs respond to a functional Dot/Icm system, and injection of LamA in particular, through activation and pathogen restriction, which is counter-evolutionary to pathogen-host adaptation.

## RESULTS

### Translocation of LamA triggers rapid glycogenolysis in PMNs.

**FIG 1:**
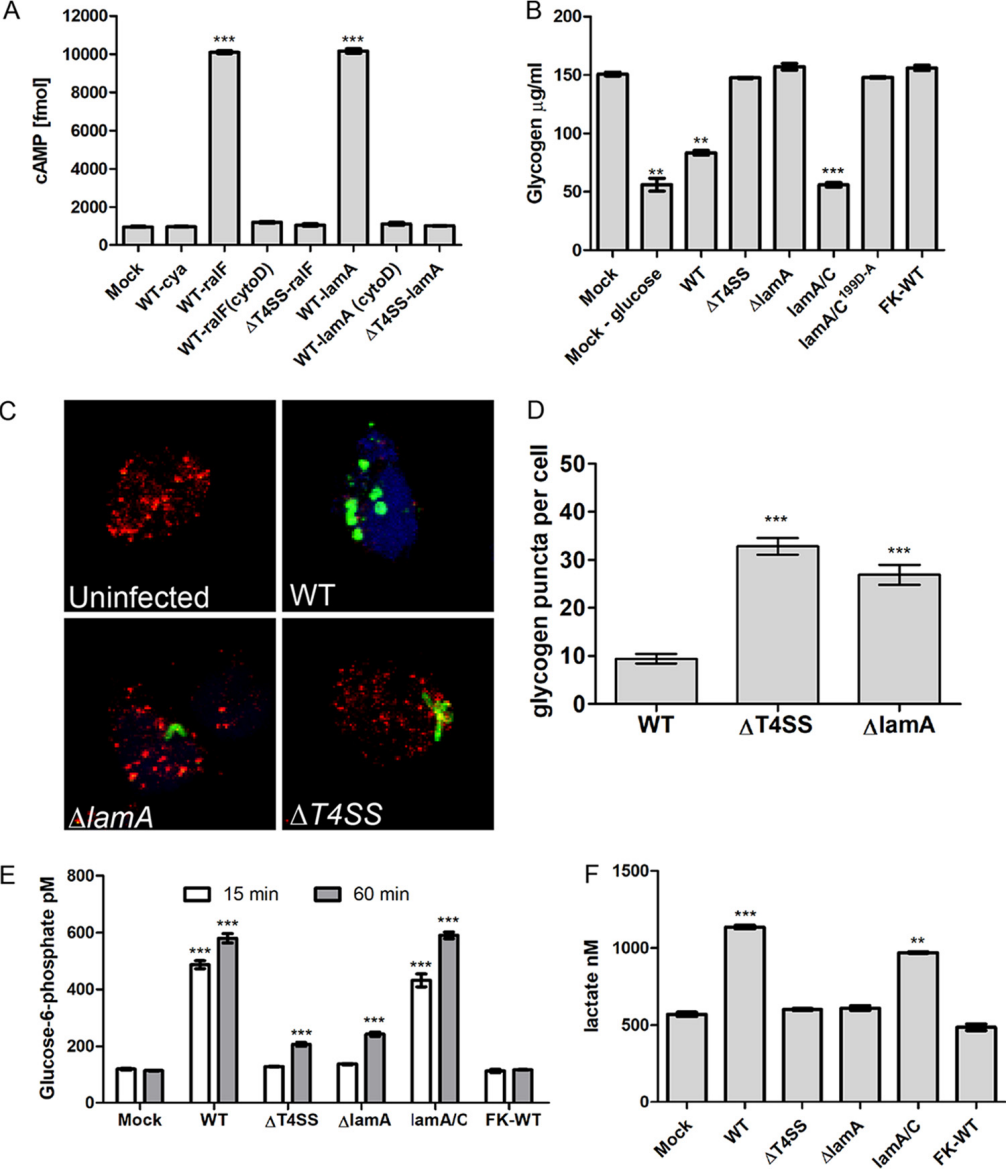
Dot/Icm-injected LamA triggers glycogenolysis in PMNs, resulting in cytosolic hyperglucose and upregulated glycolysis. (A) Adenylate cyclase (Cya) reporter fusion translocation assays of LamA expressed by wild-type L. pneumophila and the translocation-deficient Δ*T4SS* mutant. The Cya-RalF effector fusion was used a positive control. PMNs were infected for 1 h in triplicate, and cAMP production was assessed by ELISA. To prevent phagocytosis, cytochalasin D was used. Data are shown as mean cAMP concentration ± SD, *n* = 3. ***, Student's *t* test of WT-RalF or WT-LamA versus WT-Cya; *P* < 0.0001. (B) Quantification of cytosolic glycogen concentrations in PMNs starved of glucose or infected wild type, Δ*T4SS*, Δ*lamA* or *lamA/C* and its catalytically inactive mutant, and formalin-killed wild-type bacteria at 1 h postinfection. Data are shown as mean glycogen concentration ± SD; *n* = 3. **, **, and ***, Student's *t* test of mock minus glucose cells, WT, or *lam/C* versus mock-treated cells; *P* = 0.0037, *P* = 0.0014, and *P* = 0.0007, respectively. (C) Representative z-stack confocal microscopy images of PMNs infected with various L. pneumophila strains (green) and glycogen granules were labeled by antibody (red). (D) Quantification of glycogen granules per cell at 15 min postinfection. Glycogen granules were counted in z-stack confocal images, and data points show mean granules/infected cell ± SD (*n* = 100 infected cells) and are representative of three independent experiments. ***, Student's *t* test of glycogen granules in either Δ*T4SS*- or Δ*lamA*-infected cells versus wild type-infected cells; *P* < 0.0001. (E) Quantification of cytosolic glucose-6-phosphate levels in PMNs infected with L. pneumophila strains at 15 and 60 min postinfection. Data are shown as mean glucose-6-phosphate concentration ± SD, *n* = 3. ***, Student's *t* test of glucose-6-phosphate levels in either wild type- or *lamA/C*-infected cells versus uninfected cells; *P* < 0.0001. (F) Lactate levels in cell culture supernatants of PMNs infected for 1 h with wild type, Δ*T4SS*, Δ*lamA, lamA/C*, or formalin-killed wild type. Data represent the mean lactate concentration ± SD, *n* = 3. *** and **, Student's *t* test of lactate level in the culture supernatant of wild type or *lamA/C*-infected PMNs versus mock-infected cells; *P < *0.001 and *P < *0.0013, respectively. Unless otherwise stated, *n* represents technical replicates, and data shown are representative of three independent biological replicates.

Since PMNs possess abundant glycogen stores and are highly glycolytic, we determined if LamA is translocated into PMNs during infection by L. pneumophila utilizing strains harboring LamA-adenylate cyclase reporters. At 60 min postinfection, PMNs were lysed, and cAMP levels were measured by enzyme-linked immunosorbent assay (ELISA). The data showed that at 60 min postinfection, wild-type bacteria translocated LamA into the PMNs cytosol similar to the positive-control effector, RalF, but the *dotA* translocation-deficient mutant (Δ*T4SS*) failed to translocate LamA (Student's *t* test, *P* < 0.0001) (Fig. 1A). When phagocytosis of L. pneumophila by PMNs was blocked by cytochalasin D, translocation of LamA was not detected by attached extracellular wild-type bacteria (Fig. 1A), demonstrating that LamA was only translocated into the PMNs’ cytosol upon phagocytosis of L. pneumophila. Together, this indicates that LamA is rapidly injected into the PMNs’ cytosol by the Dot/Icm T4SS of intracellular L. pneumophila.

To determine the effects of the injected LamA on PMNs glycogen stores in PMNs, glycogen granules were quantified by confocal microscopy using a glycogen-specific antibody. Uninfected PMNs harbored ∼30 distinct glycogen granules. At 15 min postinfection, PMNs harboring the wild-type strain contained <10 glycogen granules (Student's *t* test, *P < *0.0001) (Fig. 1C and D). In contrast, PMNs infected with either the Δ*lamA* or Δ*T4SS* mutants harbored similar numbers of glycogen granules as uninfected PMNs (Fig. 1C and D). Therefore, the injected LamA catalyzes glycogenolysis in PMNs.

To confirm the effects of injected LamA on PMNs on glycogenysis, we determined the levels of glycogen. The PMNs were infected for 1 h with either the wild type, Δ*T4SS* or Δ*lamA* mutants, the catalytically inactive *lamA/C^199D-A^* mutant, the *lamA/C* complemented strain, or formalin-killed wild-type bacteria, and the level of glycogen was determined. As expected, control PMNs that were starved of glucose, glycogen was rapidly depleted within 1 h, indicative of glycogenolysis (Fig. 1B). Strikingly, at 1 h, glycogen was significantly depleted in PMNs infected by the wild-type strain of L. pneumophila compared to mock-infected cells (Student's *t* test, *P < *0.0014) (Fig. 1B). In contrast, glycogen levels were unaffected in PMNs infected with the Δ*T4SS* mutant, the Δ*lamA* mutant, the catalytically inactive *lamA* mutant, or the formalin-killed wild type, similar to mock-infected cells (Fig. 1B). Therefore, the catalytic activity of LamA is essential for glycogen degradation in PMNs.

### LamA-dependent cytosolic hyperglucose and enhanced glycolysis in PMNs.

Glycogen degradation/glycogenolysis is highly regulated in mammalian cells to prevent a sudden rapid rise in intracellular glucose. To determine if the unregulated and rapid LamA-mediated glycogenolysis in PMNs results in elevation of cytosolic glucose, the level of glucose-6-phosphate (G6P) in infected PMNs was determined. Following 15 min and 60 min infection, G6P levels in wild type- or *lamA/C*-infected PMNs increased ∼5-fold compared to mock-infected cells (Student's *t* test, *P < *0.0001) (Fig. 1E). In contrast, at 15 min, G6P levels in PMNs infected with the Δ*T4SS* or Δ*lamA* mutants, or formalin-killed wild-type bacteria, were similar to mock-infected cells (Fig. 1E). At 60 min, PMNs infected with the Δ*T4SS* or Δ*lamA* mutants showed slightly elevated G6P levels compared to mock-infected cells, but the increase was much less than cells infected with the wild type or the complemented *lamA/C* strain (Student's *t* test, *P < *0.0001) (Fig. 1E).

Since LamA-mediated glycogenolysis in PMNs led to cytosolic hyperglucose, we determined whether PMNs infected by the wild-type bacteria responded to the cytosolic hyperglucose by undergoing immunometabolic reprograming that involved increased aerobic glycolysis. The PMNs were infected, and the secretion of lactate into the cell culture medium at 1 h postinfection was used as a readout. Infection of PMNs with the wild-type strain resulted in a significant increase in lactate secretion into the cell culture media compared to mock-infected cells (Student's *t* test, *P* < 0.001) (Fig. 1F). In contrast, PMNs infected with the Δ*T4SS* or Δ*lamA* mutants or formalin-killed wild-type bacteria produced similar amounts of lactate to mock-infected cells (Fig. 1F). Importantly, infection of PMNs with the complemented Δ*lamA/C* mutant resulted in increased lactate secretion, similar to infection with the wild-type strain (Fig. 1F). This indicates that in response to LamA-dependent cytosolic hyperglucose, PMNs exhibit immunometabolic reprograming that involved the hallmark of increased glycolysis.

### LamA-dependent rapid degradation of L. pneumophila within PMNs.

Permissiveness of human macrophages or the natural amoebal hosts to L. pneumophila is totally dependent on a functional Dot/Icm T4SS apparatus since the Δ*T4SS* mutant is degraded within macrophages or amoebae. Strikingly, confocal microscopic analyses of L. pneumophila morphology revealed that the wild-type bacteria became rounded and fragmented within 15 min of infection (Fig. 1C), while the Δ*T4SS* and Δ*lamA* mutants remained intact bacillus-shaped bacteria (Fig. 1C). This suggested PMNs actively degraded intracellular wild-type bacteria but not the Δ*T4SS* and Δ*lamA* mutants. Under the infection conditions, no evidence for neutrophil extracellular traps was observed.

**FIG 2:**
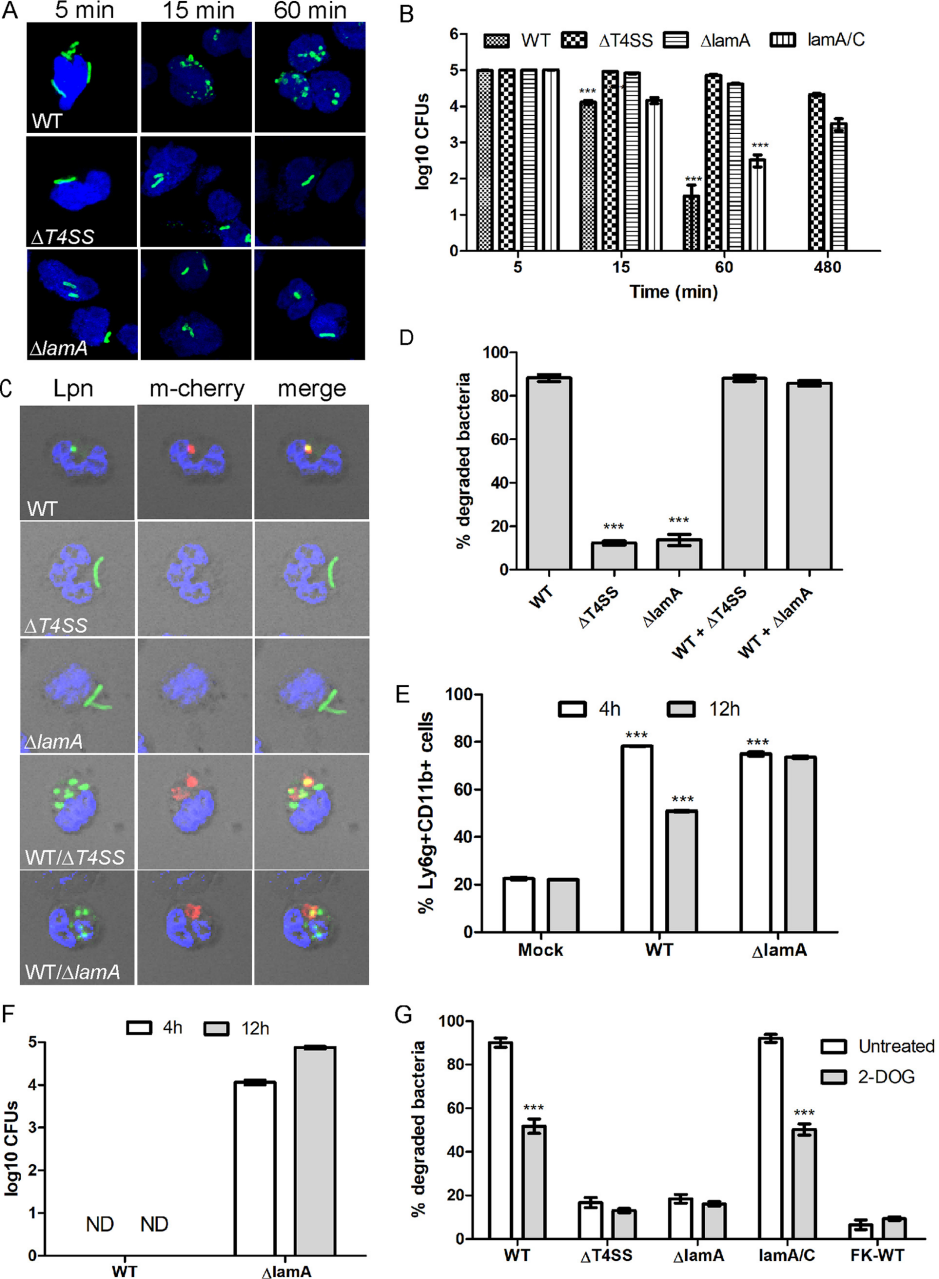
Dot/Icm- and LamA-dependent rapid killing of L. pneumophila by PMNs. (A) Representative confocal images of PMNs infected with either the wild type or the Δ*T4SS* or Δ*lamA* mutants at 5, 15, and 60 min postinfection. Bacteria are labeled with an anti-*Legionella* antibody (green), and nuclei are stained with DAPI (blue). (B) Survival of L. pneumophila in PMNs was determined by measuring recoverable CFU from infected cells. PMNs were infected with the wild type or the Δ*T4SS* or Δ*lamA* mutants or the complemented *lamA/C* mutant, and CFU were determined at 5, 15, 60, and 480 min postinfection. Data are shown as mean log_10_ CFU ± SD; *n* = 3. ***, Student's *t* test CFU at 15 and 60 min relative to 5 min; *P *< 0.0001. (C and D) Degradation of the Δ*T4SS* or Δ*lamA* mutants in PMNs when coinhabiting the same cell with wild-type bacteria. PMNs were infected with the wild type expressing mCherry alone (red), the Δ*T4SS* or Δ*lamA* mutants alone, or with wild type coinfected with Δ*T4SS* or Δ*lamA* mutants. Following 1 h infection, bacterial morphology was examined by confocal microscopy. Representative confocal images are shown in panel C and quantification in panel D. Data are shown as mean percent degraded bacteria ± SD; *n* = 100, ***, Student's *t* test of percent degraded Δ*T4SS* or Δ*lamA* mutants in PMNs versus wild-type bacterial cells; *P* < 0.0001. (E) Analysis of Ly6g^+^ CD11b^+^ PMN infiltration in lungs of mock-infected mice or infected with either the wild type or Δ*lamA* mutant at 4 h and 12 h postinfection. (F) Survival of the wild type or the Δ*lamA* mutant in Lyg6^+^ CD11b^+^ PMNs isolated from the lungs of infected mice. (G) Survival of L. pneumophila in PMNs during inhibition of glycolysis with 2-deoxyglucose was determined by confocal microscopy. PMNs pretreated with 2-deoxyglucose were infected with wild type or the Δ*T4SS* or Δ*lamA* mutants or formalin-killed wild type, and bacterial morphology was examined at 15 min postinfection. ***, Student's *t* test of percent degraded wild type or *lamA/C* bacteria in PMNs treated with 2-deoxyglucose versus untreated cells; *P* < 0.0001. Unless otherwise stated, *n* represents technical replicates, and data shown are representative of three independent biological replicates.

To assess the kinetics of degradation of the wild-type bacteria, PMNs were examined by single-cell analyses using confocal microscopy at 5, 15, and 60 min postinfection. At 5 min postinfection, the morphology of the wild-type strain and both mutants showed no alterations with normal intact bacillus-shaped bacteria. Importantly, the Δ*T4SS* and Δ*lamA* mutants retained normal bacterial morphology up to 60 min postinfection (Fig. 2A). In contrast, at 15 min, most wild-type bacteria with the functional Dot/Icm T4SS apparatus became rounded and fragmented (Fig. 2A). Viability of the Δ*lamA* was compromised, similar to the wild-type strain, when complemented by the native *lamA* gene (Δ*lamA/C*) but not by the catalytically inactive Δ*lamA/C^D199A^/^E233A^/^D313A^* mutants (Fig. S1 in the supplemental material).

10.1128/mBio.01008-21.1 FIG S1 Dot/Icm- and LamA-dependent rapid killing of L. pneumophila by PMNs. (A) Representative confocal images of PMNs infected with the Δ*lamA* mutant complemented with the wild-type lamA gene or the catalytically inactive Δ*lamA/C^D199A^/^E233A^/^D313A^* strain 15 and 60 min postinfection. Bacteria are labeled with an anti-*Legionella* antibody (green), and nuclei are stained with DAPI (blue). Download FIG S1, TIF file, 1.1 MB. Copyright © 2021 Price et al. 2021 Price et al. https://creativecommons.org/licenses/by/4.0/ This content is distributed under the terms of the Creative Commons Attribution 4.0 International license.

To confirm that the wild-type bacteria but not the Δ*T4SS* or Δ*lamA* mutants were degraded by the PMNs, we examined viability of bacteria by enumerating CFU from infected cells at various time points. At 5 min postinfection, all strains showed similar CFU and are representative of the initial inoculums (Fig. 2B). At 15 and 60 min postinfection, the Δ*T4SS* and Δ*lamA* mutants showed no reduction in viability (Fig. 2B). In addition, the Δ*T4SS* and Δ*lamA* mutants showed little decrease in viable bacteria, even at 8 h postinfection (Fig. 2B). Remarkably, by 15 min postinfection, the wild-type strain and *lamA/C* showed ∼10-fold reductions in viability (Student's *t* test, *P < *0.0001). By 1 h, a further ∼400-fold reduction in CFU was observed for the wild-type strain (Student's *t* test, *P < *0.0001) (Fig. 2B). No viable wild-type strain or *lamA/C* were detected at 8 h postinfection (Fig. 2B).

Previous data have shown that opsonization with both antibody and complement is required for efficient association and uptake of L. pneumophila by PMNs (13, 89). Therefore, we determined whether the observed differences in wild-type survival in PMNs compared to the Δ*T4SS* mutant strain were due to differences in bacterial opsonization and uptake. At 15 min postinfection, uptake of both wild type and the Δ*T4SS* mutant were similar when opsonized with either fresh human serum or human serum plus anti-*Legionella* antibody (Fig. S2A). Consistent with previous observations, nonopsonized wild-type bacteria were poorly taken up by PMNs at 15 min postinfection, while uptake of nonopsonized Δ*T4SS* was similar to the opsonized mutant (Fig. S2A). Regardless of opsonization, within 60 min postinfection, both wild-type bacteria and Δ*T4SS* mutant bacteria were taken up by PMNs at similar levels (Fig. S2A). Although formalin-killed bacteria generally showed lower uptake by PMNs compared to wild-type and Δ*T4SS* mutant bacteria, there was no statistically significant difference (Fig. S2A).

10.1128/mBio.01008-21.2 FIG S2 Impact of opsonization on L. pneumophila uptake and bacterial survival by PMNs. (A) Uptake of wild type, formalin-killed wild type (FK-WT), and the Δ*T4SS* mutant was assessed at 15 and 60 minutes postinfection. Prior to infection, bacteria were unopsonized (mock) or opsonized with human serum alone or human serum plus anti-*Legionella* antibody. Data shown are percentage of bacteria taken up by PMNs ± SD (*n* = 100 PMNs) performed with three technical replicates and are representative of 3 independent biological replicates. ** and ***, Student *t*-test of wild-type uptake of human serum or human serum plus antibody opsonized versus unopsonized; *P* = 0.0035 and = 0.0008, respectively. (B) Analysis of bacterial morphology of wild type, formalin-killed wild type (FK-WT), and the Δ*T4SS* mutant was assessed at 15 and 60 minutes postinfection. Bacteria were opsonized as described above. Data show percentage of bacteria with altered morphology ± SD (*n* = 100 bacteria) performed with three technical replicates and are representative of 3 independent biological replicates. **, Student *t*-test of wild type uptake of human serum opsonized versus unopsonized; *P* = 0.0016. Download FIG S2, TIF file, 0.5 MB. Copyright © 2021 Price et al. 2021 Price et al. https://creativecommons.org/licenses/by/4.0/ This content is distributed under the terms of the Creative Commons Attribution 4.0 International license.

Next, we examined if bacterial degradation was altered by opsonization. The data showed that the majority of wild-type bacteria opsonized with human serum or human serum plus anti-*Legionella* antibody were degraded at 15 min postinfection, while most Δ*T4SS* mutant bacteria remained intact (Fig. 2B). At 60 min postinfection, there was an increase in degradation of the Δ*T4SS* mutant bacteria, but this was not statistically significant. As expected, minimal degradation of formalin-killed wild-type bacteria was observed at 15 and 60 min postinfection regardless of opsonization (Fig. 2B). We conclude that both opsonized wild-type and Δ*T4SS* bacteria were taken up similarly by PMNs (Fig. 2A), and the degradation of wild-type bacteria is not due to differences in uptake and opsonization compared to the Δ*T4SS* mutant bacteria. Thus, in contrast to the Dot/Icm-dependent permissiveness of macrophages to L. pneumophila, PMNs rapidly degrade wild-type L. pneumophila in a Dot/Icm-dependent manner, and specifically, the LamA effector is essential for rapid restriction of L. pneumophila by PMNs.

Next, we determined if cohabitation of wild-type bacteria with the Δ*T4SS* or Δ*lamA* mutants within the same PMN resulted in rapid killing of the mutants. To achieve this, PMNs were infected with either the wild type expressing mCherry or the Δ*T4SS* and Δ*lamA* mutants individually or coinfected with the wild type expressing mCherry and the Δ*T4SS* or Δ*lamA* mutants together. Following 1 h infection, bacterial morphology in infected PMNs was examined by confocal microscopy (Fig. 2C and D). In PMNs infected with wild-type bacteria alone, ∼90% of bacteria showed deformed morphology, while only 12% and 14% of Δ*T4SS* or Δ*lamA* bacteria showed deformed morphology (Fig. 2C and D). In contrast, in PMNs coinfected with both wild type and the Δ*T4SS* or Δ*lamA* mutants, 90% of all bacteria exhibited deformed morphology (Student's *t* test, *P < *0.0001) (Fig. 2C and D). This indicates that, in response to injection of LamA by wild-type bacteria, PMNs are activated and restrict the coinhabiting Δ*T4SS* or Δ*lamA* mutants.

To confirm the LamA-dependent rapid killing of L. pneumophila by PMNs in an *in vivo* infection model, A/J mice were infected with either the wild-type strain or the Δ*lamA* mutant and PMNs collected at 4 h and 12 h postinfection by bronchoalveolar lavage (BAL) fluid. At 4 h and 12 h postinfection, BAL fluid was retrieved from infected mice and analyzed by flow cytometry to determine the PMN Ly6g-positive (Ly6g^+^) CD11b^+^ cell population. The data showed a dramatic influx of Lyg6^+^ CD11b^+^ cells at 4 h in both wild-type and Δ*lamA*-infected mice compared to mock-infected mice (Student's *t* test, *P < *0.0001) (Fig. 2E). At 12 h postinfection, the number of Ly6g^+^ CD11b^+^ cells fell to 50% in wild type-infected mice (Student's *t* test, *P < *0.0001), but no reduction was seen in Δ*lamA*-infected mice (Fig. 2E). Next, we determined recoverable bacterial CFU in the PMNs sorted from the BAL fluid. In wild type-infected mice, no recoverable CFU were obtained in the Ly6g+ CD11b+ PMNs population. In contrast, ∼4- to 5-log_10_ CFU of the Δ*lamA* mutant were recovered from the sorted PMNs (Fig. 2F). This indicates that similar to *ex vivo* in human PMNs, wild-type bacteria are rapidly cleared by PMNs *in vivo*, and this killing is LamA dependent.

### Enhanced glycolysis-mediated activation of PMNs in response to cytosolic hyperglucose.

We examined if increased glycolysis in response to LamA-dependent cytosolic hyperglucose resulted in PMNs activation and rapid killing of L. pneumophila. The PMNs were pretreated with the glycolysis inhibitor 2-deoxyglucose (2-DOG), and infected and bacterial morphology were examined by confocal microscopy. Prolonged exposure to 2-DOG is toxic to L. pneumophila (90), but the 15 min exposure of L. pneumophila to this inhibitor did not impair viability (Fig. S3). In mock-infected PMNs, ∼90% of wild-type bacteria exhibited deformed morphology. However, in PMNs where glycolysis was inhibited, only ∼50% of wild-type bacteria showed deformed morphology (Student's *t* test, *P < *0.0001) (Fig. 2G). In PMNs infected with the Δ*T4SS* or Δ*lamA* mutants or formalin-killed wild-type bacteria, most bacteria maintained normal morphology, and inhibition of glycolysis did not impact this (Fig. 2G). Importantly, PMNs infected with the complemented *lamA/C* mutant, ∼90% of the bacteria exhibited deformed morphology, but in PMNs where glycolysis was inhibited, only ∼50% showed deformed morphology (Student's *t* test, *P < *0.0001) (Fig. 2G). This indicates that PMNs respond to cytosolic hyperglucose generated by LamA-dependent glycogenolysis and increased glycolysis leading to activation of PMNs and restriction of L. pneumophila.

10.1128/mBio.01008-21.3 FIG S3 Bacterial viability of L. pneumophila in the presence of 2-deoxyglucose. To assess viability of L. pneumophila in the presence of 2-DOG, bacteria were incubated with and without the inhibitor for 15 minutes, and CFU were determined. Download FIG S3, TIF file, 0.1 MB. Copyright © 2021 Price et al. 2021 Price et al. https://creativecommons.org/licenses/by/4.0/ This content is distributed under the terms of the Creative Commons Attribution 4.0 International license.

### Spatial ROS production by PMNs within the LCP.

**FIG 3:**
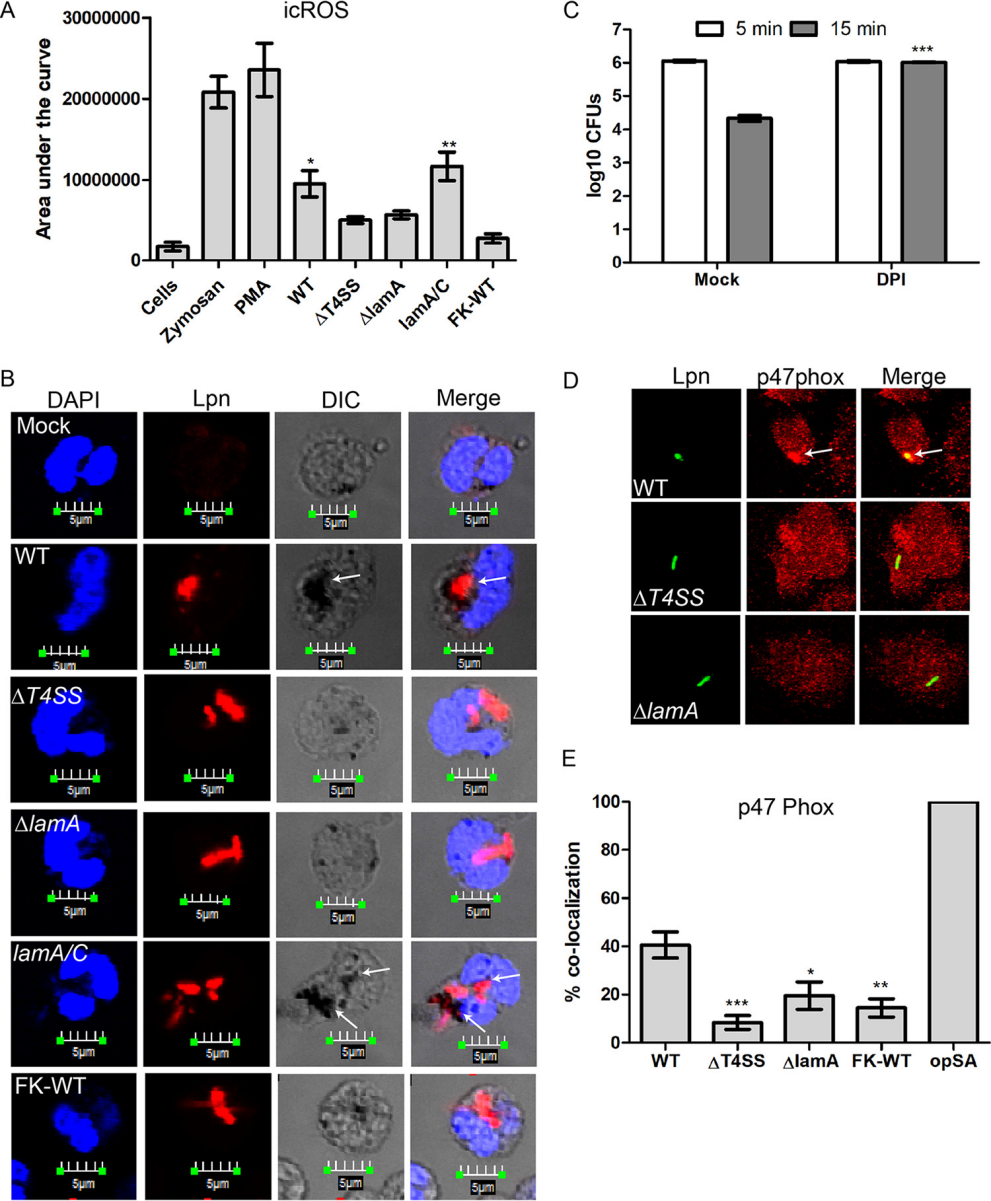
Generation of a robust ROS burst by PMNs in response to cytosolic hyperglucose generated by LamA-mediated glycogenolysis. (A) Determination of ROS produced by PMNs in response to L. pneumophila infection. PMNs were infected with either the wild-type bacteria or the Δ*T4SS* and Δ*lamA* mutants, the complemented *lamA/C* mutant, or formalin-killed wild type for 1 h. Additionally, PMNs were stimulated with zymosan or PMA as a positive control for ROS production. *, Student's *t* test of ROS production by PMNs infected with either Δ*T4SS* or the Δ*lamA* mutant versus wild type infected PMNs; *P* = 0.0277, 0.0432, respectively. (B) Representative confocal microscopy images showing spatial subcellular localization of ROS production in the pathogen containing phagosome. PMNs treated with NBT were infected with wild type or the Δ*T4SS* or Δ*lamA* mutants, the complement *lamA/C* mutant, or formalin-killed wild type for 1 h. Bacteria were stained using an anti-L. pneumophila antibody (red), and nuclei were stained with DAPI (blue). NBT staining is shown in differential interference contrast (DIC) images as black deposits. (C) The contribution of ROS to rapid bacterial killing was determined using the inhibitor DPI. PMNs were pretreated with DPI and infected with wild-type bacteria, and recoverable CFU were determined at 5 and 15 min postinfection. Data are shown as mean log_10_ CFU ± SD; *n* = 3. ***, Student's *t* test of wild-type CFU in DPI-treated PMNs at 15 min versus untreated PMNs; *P* < 0.0001. (D) Representative confocal images showing spatial subcellular colocalization of p47phox with the L. pneumophila phagosome. PMNs were infected with wild type or the Δ*T4SS* or Δ*lamA* mutants for 15 min. Bacteria are stained with an anti-*Legionella* antibody (green), and p47phox is stained with anti-p47phox (red). (E) Quantification of p47phox colocalization to the pathogen-containing phagosome at 15 min postinfection. Data are shown as mean percent colocalization ± SD; *n* = 100. ***, *, and **, Student's *t* test of percent colocalization of p47phox to phagosomes harboring the Δ*T4SS* or Δ*lamA* mutants, formalin-killed wild type, or opsonized S. aureus versus wild type containing phagosomes; *P* = 0.0008, = 0.0288, and = 0.0043, respectively. Unless otherwise stated, *n* represents technical replicates, and data shown are representative of three independent biological replicates.

One of the two major antimicrobial arms of the PMN response to bacterial infection is the oxygen-dependent generation of ROS by the phagocyte NADPH oxidase. Since L. pneumophila was rapidly killed by PMNs, we determined if PMNs generated intracellular ROS (icROS) in response to L. pneumophila infection (91). Control PMNS were stimulated with PMA or zymosan as a positive control for icROS production. The data showed that in response to infection with the wild-type strain or the complemented *lamA/C* mutant, PMNs produced significantly more icROS (Student's *t* test, *P* = 0.0104 and = 0.0058, respectively) than cells infected with the Δ*T4SS* or Δ*lamA* mutants or formalin-killed wild-type bacteria (Fig. 3A).

To determine if the increased icROS production in PMNs occurred spatially within the LCP, PMNs were stained using nitroblue tetrazolium (NBT), which forms dense deposits in the presence of ROS. The data showed that phagosomes harboring either the wild-type strain or the complemented *lamA/C* mutant colocalized with dense deposits of NBT, but little or no NBT staining was seen in PMNs infected with the Δ*T4SS* or Δ*lamA* mutants or formalin-killed wild-type bacteria (Fig. 3B). Importantly, NBT staining was spatial and limited subcellularly to the LCP (Fig. 3B).

Since ROS was spatially generated within the wild-type (WT) strain LCP, we determined if ROS contributes to the rapid killing of wild-type bacteria. To determine this, PMNs were pretreated with the ROS inhibitor diphenyleneiodonium (DPI) prior to infection, and recoverable bacterial CFU were enumerated. At 5 min postinfection, similar numbers of CFU were recovered from mock- and DPI-treated PMNs (Fig. 3C). However, at 15 min, ∼100-fold fewer wild-type bacteria were recovered from mock-treated PMNs (Fig. 3C). In contrast, in DPI-treated PMNs, no significant reduction in recoverable CFU was observed (Student's *t* test, *P < *0.0001) (Fig. 3C). Our data indicate that spatial generation of ROS within LCP plays a key role in the rapid killing of wild-type bacteria in PMNs.

### Acquisition of the phagocyte NADPH oxidase by the LCP.

Since PMNs produced increased ROS in response to wild-type strain infection and this ROS is spatially produced within the LCP, we examined recruitment of the NADPH oxidase cytosolic component p47phox to the LCP at 5, 15, and 60 min postinfection (Fig. 3D and E; Fig. S4A). Opsonized Staphylococcus aureus was used as a positive control for recruitment of the NADPH oxidase. As early as 5 min postinfection, ∼33% of wild-type LCPs colocalized with p47phox (Fig. S4A), while LCPs harboring the Δ*T4SS* or Δ*lamA* mutants or formalin-killed wild-type bacteria showed significantly less colocalization with p47phox (Fig. S4A). At 15 min postinfection, ∼40% of wild-type LCPs colocalized with p47phox (Fig. 3D and E). In contrast, ∼20% or fewer phagosomes harboring the Δ*T4SS* or Δ*lamA* mutants, or formalin-killed wild-type bacteria colocalized with p47phox (Student's *t* test, *P < *0.0008, *P < *0.0288, and *P < *0.0043, respectively) (Fig. 3D and E). Following 1 h of infection, localization of p47phox to wild-type LCPs increased to ∼50%, while LCPs harboring the Δ*T4SS* or Δ*lamA* mutants, or formalin-killed wild-type bacteria, were still below 20% (Student's *t* test, *P < *0.0001) (Fig. S4A). Taken together, these indicate that the phagocyte NADPH oxidase complex is spatially targeted to the wild-type strain containing phagosomes in a Dot/Icm- and LamA-dependent manner.

10.1128/mBio.01008-21.4 FIG S4 LamA-dependent fusion of specific and azurophilic granules and NADPH oxidase to the L. pneumophila phagosome. (A) Quantification of p47phox colocalization to the L. pneumophila phagosome at 5 and 60 min postinfection. Data are shown as mean percent colocalization ± SD; *n* = 100. ***, Student *t*-test of percentage of colocalization of p47 to phagosomes harboring the Δ*T4SS* or Δ*lamA* mutants or formalin-killed wild type versus wild type containing phagosomes; *P < *0.0001. (B) Quantification of lactoferrin colocalization to the L. pneumophila phagosome at 5 and 60 min postinfection. Data are shown as mean percent colocalization ± SD; *n* = 100. ***, Student *t*-test of percent colocalization of lactoferrin to phagosomes harboring the Δ*T4SS* or Δ*lamA* mutants or formalin-killed wild type versus wild type containing phagosomes; *P < *0.0001. (C) Quantification of elastase colocalization to the L. pneumophila phagosome at 5 and 60 min postinfection. Data are shown as mean percent colocalization ± SD; *n* = 100. ***, Student *t*-test of percent colocalization of lactoferrin to phagosomes harboring the Δ*T4SS* or Δ*lamA* mutants or formalin-killed wild type versus wild type containing phagosomes; *P < *0.0001. Unless otherwise stated, *n* represents technical replicates, and data shown are representative of three independent biological replicates. Download FIG S4, TIF file, 0.7 MB. Copyright © 2021 Price et al. 2021 Price et al. https://creativecommons.org/licenses/by/4.0/ This content is distributed under the terms of the Creative Commons Attribution 4.0 International license.

### LamA-dependent fusion of neutrophil granules to the LCP.

The other major arm of the PMNs microbicidal activity is the fusion of specific and azurophilic granules to bacteria-containing phagosomes. Therefore, we determined whether the antimicrobial matrix contents of specific and azurophilic granules were delivered to the LCP. Within 5 min, ∼40% of wild type containing phagosomes colocalized with lactoferrin and elastase, markers for specific and azurophilic granules, respectively, similar to the opsonized S. aureus control (Fig. S4B and C). In contrast, less than 10% of phagosomes containing either the Δ*T4SS* or Δ*lamA* mutants or formalin-killed wild-type bacteria colocalized to either lactoferrin or elastase (Student's *t* test, *P < *0.0001) (Fig. S4B and C).

**FIG 4:**
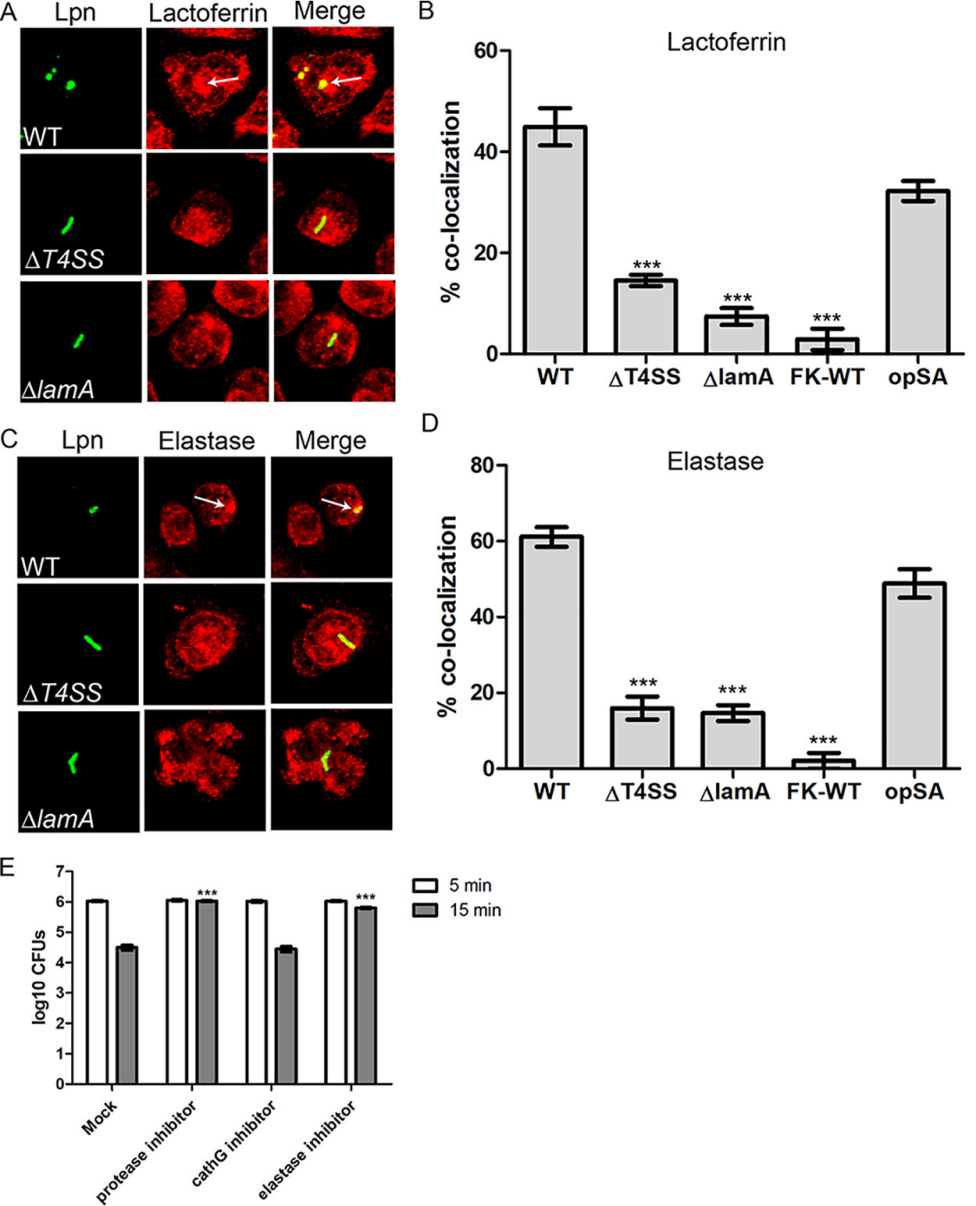
LamA-dependent fusion of specific and azurophilic granules to the L. pneumophila phagosome. (A) Representative confocal images showing colocalization of lactoferrin with the L. pneumophila phagosome. PMNs were infected with wild type or the Δ*T4SS* or Δ*lamA* mutants, formalin-killed wild type, or opsonized S. aureus for 15 min. Bacteria are stained with an anti-*Legionella* antibody (green), and lactoferrin is stained with anti-lactoferrin (red). (B) Quantification of lactoferrin colocalization to the L. pneumophila phagosome at 15 min postinfection. Data are shown as mean percent colocalization ± SD; *n* = 100. ***, Student's *t* test of percent colocalization of lactoferrin to phagosomes harboring the Δ*T4SS* or Δ*lamA* mutants or formalin-killed wild type versus wild type containing phagosomes; *P < *0.0001. (C) Representative confocal images showing colocalization of elastase with the L. pneumophila phagosome. PMNs were infected with wild type or the Δ*T4SS* or Δ*lamA* mutants, formalin-killed wild type, or opsonized S. aureus for 15 min. Bacteria were labeled with an anti-*Legionella* antibody (green), and elastase is labeled with anti-elastase antibodies (red). (D) Quantification of elastase colocalization to the L. pneumophila phagosome at 15 min postinfection. Data are shown as mean percent colocalization ± SD; *n* = 100. ***, Student's *t* test of percent colocalization of elastase to phagosomes harboring the Δ*T4SS* or Δ*lamA* mutants or formalin-killed wild type versus wild type containing phagosomes; *P < *0.0001. (E) Relative contribution of specific and azurophilic granules to rapid bacterial killing was determined using a pan-protease, cathepsin G, and elastase inhibitors. PMNs were pretreated with DPI and infected with wild-type bacteria, and recoverable CFU were determined at 5 and 15 min postinfection. Data are shown as mean log_10_ CFU ± SD; *n* = 3. ***, Student's *t* test of wild-type CFU in PMNs treated with the pan-protease or elastase inhibitors at 15 min versus untreated PMNs; *P *< 0.0001. Unless otherwise stated, *n* represents technical replicates, and data shown are representative of three independent biological replicates.

At 15 min postinfection, ∼40% of wild-type LCPs still colocalized with lactoferrin, but colocalization with elastase increased to ∼60%, similar to the opsonized S. aureus control, while less than 15% of phagosomes containing either the Δ*T4SS* or Δ*lamA* mutants or formalin-killed wild-type bacteria colocalized to either lactoferrin or elastase (Student's *t* test, *P < *0.0001) (Fig. 4A to D). At 1 h postinfection, colocalization of lactoferrin with phagosomes harboring wild-type bacteria diminished to ∼24% compared to ∼44% for the opsonized S. aureus control (Fig. S4B). However, colocalization of lactoferrin to LCPs containing the Δ*T4SS* or Δ*lamA* mutants, or formalin-killed wild-type bacteria, was still lower than that observed for wild type containing phagosomes but not statistically significant (Fig. S4B). In contrast, at 1 h postinfection, 67% of LCPs harboring the wild-type strain still colocalized with elastase, similar to the opsonized S. aureus control (Fig. S4C). However, LCPs containing either the Δ*T4SS* or Δ*lamA* mutants or formalin-killed wild-type bacteria still showed significantly lower colocalization with elastase than the wild-type strain (Student's *t* test, *P < *0.0001) (Fig. S4C).

Since both specific and azurophilic granules fused to the wild type containing LCPs, we determined if these granules contribute to the rapid killing of wild-type bacteria in addition to ROS. To determine this, PMNs were either pretreated with a pan-protease inhibitor, a cathepsin G inhibitor (specific granules), or an elastase inhibitor (azurophilic granules) and infected with wild-type bacteria for 5 and 15 min, and recoverable CFU were enumerated. At 5 min postinfection, similar numbers of wild-type CFU were recovered from PMNs treated with the inhibitors compared to mock-treated cells (Fig. 4E). At 15 min postinfection, recoverable CFU were reduced by ∼100-fold in mock-treated PMNs. In contrast, in PMNs treated with the pan-protease inhibitor or the elastase inhibitor, wild-type bacterial viability was not altered at 15 min postinfection (Student's *t* test, *P < *0.0001) (Fig. 4E). In PMNs treated with the cathepsin G inhibitor, recoverable wild-type CFU were similar to mock-treated cells (Fig. 4E), indicating that blocking specific granules had little or no effect on the ability of PMNs to restrict wild-type bacteria. Taken together, fusion of azurophilic granules to the LCP along with spatial ROS generation within the LCP contribute to the rapid restriction of L. pneumophila. Pathogen restriction is dependent on immunometabolic reprogramming and activation of the PMNs in response to cytosolic hyperglucose generated by rapid and uncontrolled LamA-mediated unregulated glycogenolysis.

## DISCUSSION

PMNs represent key immune cells in the innate defense against bacterial pathogens. Many human intracellular bacterial pathogens have undergone selective evolutionary pressure and adaptation to the hosts they invade to interfere with the inflammatory response and innate defenses of macrophages and PMNs to override pathogen restriction in these phagocytic cells (92, 93). In contrast to the general theme of pathogens being specialists in terms of their host range, L. pneumophila is a generalist that resides in a diverse range of amoebae species in the environment and has acquired a large repertoire of effector proteins to survive and proliferate within these host cells (6). Indeed, deletion of one-third of the known L. pneumophila effectors does not impact the ability of L. pneumophila to replicate in mouse-derived macrophages (94). It is thought that the large arsenal of L. pneumophila effectors with an unusually high level of redundancy represents a toolbox for adaptation to various environmental hosts (6). Even though L. pneumophila can infect and proliferate in human macrophages, causing an accidental pneumonia, this bacterial pathogen has not evolved strategies to effectively overcome the mammalian innate immune response of PMNs. In 1981, it was reported that PMNs restrict L. pneumophila, but the mechanisms remained unknown (55), even 40 years later. Here, we show that human PMNs rapidly kill L. pneumophila. The mechanism is mediated by the PMNs’ direct response to the cellular and biochemical effects of LamA injection through the Dot/Icm type IV secretion system. The injected LamA enzyme catalyzes rapid unregulated glycogenolysis, leading to a sudden cytosolic hyperglucose that upregulates glycolysis, which triggers PMNs activation. Paradoxically, the LamA effector results in rapid activation of PMNs, leading to L. pneumophila degradation by the PMNs’ two major bactericidal machineries, which are the spatial generation of ROS within the LCV, and fusion of azurophilic and specific granules to the phagosome, which effectively degrade L. pneumophila within 15 to 60 min postphagocytosis.

The LamA-dependent activation of PMNs may mimic hyperglycemia in diabetic patients. Hyperglycemia triggers activation of PMNs similar to *ex vivo* exposure of quiescent PMNs to high glucose (62–64), and this response is mediated through upregulation of aerobic glycolysis (62–64). However, unlike hyperglycemia, which is limited by the bottleneck of glucose uptake by the glucose transporter, LamA-mediated glycogenolysis results in an ∼5- to 6-fold rise in cytosolic glucose-6-phosphate levels. It is possible that the elevated glucose-6-phosphate levels in PMNs infected with wild-type bacteria come from both the elevated glycogenolysis observed and through increased transport of extracellular glucose. However, this is unlikely since our previous study (88) has demonstrated that the rapid rise in intracellular glucose-6-phosphate came directly from elevated glycogenolysis and not increased transport of extracellular glucose since blocking GLUT1 transport had no effect on the rapid rise of glucose levels. Due to the bottleneck of limited glucose import by the glucose transporters in the plasma membrane, this level of LamA-generated cytosolic glucose is not achieved even in severe cases of chronic hyperglycemia. This may trigger other dysregulated immunometabolic changes that impact killing of L. pneumophila (95). Although LamA performs the same catalytic activity in both human macrophages (88) and PMNs, the outcome of unregulated glycogenolysis is different since macrophages are permissive, while PMNs restrict and degrade L. pneumophila.

Since the type IV translocation deficient mutant survived in PMNs better than the *lamA* mutant, it is likely that effectors other than LamA also contribute to the ability of human PMNs to sense and then rapidly kill L. pneumophila. Furthermore, formalin- or heat-killed L. pneumophila appeared to be resistant to degradation by PMNs, which may be attributable to the inert nature of L. pneumophila LPS (96), suggesting multiple factors besides LamA contribute to the ability of human PMNs to sense and kill this pathogen, but the other mechanisms behind this remain to be elucidated.

The coevolution of L. pneumophila with its amoebal hosts has equipped this bacteria with a large and redundant arsenal of effectors to survive in these diverse and hostile hosts (6). Many of the effectors in the L. pneumophila molecular toolbox are likely specific to certain environmental hosts and are either dispensable in other hosts or have paradoxical consequences. For example, LamA is required to subvert amoebal encystation to enhance its survival in the environmental host, but in human macrophages, this effector promotes a proinflammatory response that partially restricts L. pneumophila replication (88). Even though not all effectors are required for survival in host cells, the Dot/Icm translocation system itself is essential for L. pneumophila survival in amoebae and human macrophages and therefore represents the key virulence system of this pathogen. However, when L. pneumophila encounters mammalian PMNs in the accidental dead-end human host, it is ill-equipped to subvert killing. In contrast to amoebae and macrophages, the Dot/Icm translocation system and the associated cadre of effectors injected into the host cells and, in particular, LamA, may represent an unintended antivirulence process for L. pneumophila in PMNs through triggering the two major microbicidal arms of the innate defense of PMNs. The accidental LamA-dependent hyperglucose in PMNs is responsible for their activation and rapid degradation of L. pneumophila by PMNs. These findings highlight unexpected phenotypes of amoebae-adapted effectors of L. pneumophila that have evolved to interfere with processes of the amoeba natural host with unintended paradoxical effects in the accidental human host.

## MATERIALS AND METHODS

### Strains and cell lines.

L. pneumophila strain AA100/130b (ATCC BAA-74), the T4SS-deficient mutant (Δ*T4SS*), Δ*lamA*, the complemented *lamA/C*, and the catalytically inactive mutant *lamA/C*^199D-A^ were grown on BCYE agar. For infections, bacteria were grown overnight to post-exponential phase in BYE broth culture to an optical density at 550 nm (OD_550_) of 2 to 2.2. Neutrophils were isolated from venous blood of healthy donors using plasma‐Percoll gradients (91). Recruitment of donors, as well as the blood draws, was in accordance with the guidelines approved by the institutional review board of the University of Louisville. Isolated cells showed that ≥90 to 95% were neutrophils by microscopic evaluation of cytospins and Wright staining. Trypan blue exclusion indicated that >97% of cells were viable.

### Opsonization of L. pneumophila for PMN infection.

For infection of PMNs, L. pneumophila was opsonized. A total of 500 μl of post-exponential-phase L. pneumophila broth culture was pelleted by centrifugation and resuspended in 500 μl of sterile water. A total of 1 × 10^8^ bacteria were added to 500 μl of Hanks buffered salt solution (HBSS) containing 10% pooled human serum and 1 μl of mouse anti-*Legionella* monoclonal antibody. Following 30 min incubation at room temperature, opsonized bacteria were pelleted by centrifugation and resuspended in 100 μl sterile water. For some experiments, L. pneumophila was killed using 4% (vol/vol) formaldehyde prior to opsonization. Briefly, post-exponential-phase bacteria were pelleted by centrifugation and resuspended in water containing 4% (vol/vol) formaldehyde and incubated at room temperature for 30 min. Bacteria were then washed three times with sterile water prior to opsonization.

### Translocation assay.

To assess translocation of LamA by L. pneumophila during infection of PMNs, an adenylate cyclase fusion to the N terminus of LamA (97) was used as described previously. A total of 10 × 10^6^ PMNs were infected with wild-type or Δ*T4SS* mutant L. pneumophila harboring plasmids expressing various adenylate cyclase fusions at a multiplicity of infection (MOI) of 1 for 1 h as described previously (97, 98). In some wells, to prevent phagocytosis, PMNs were pretreated with cytochalasin D prior to infection. Following infection, the cell monolayers were lysed and processed to assess cAMP concentration by ELISA using the Direct cAMP ELISA kit (Enzo) according to the manufacturer’s instructions.

### Analysis of intracellular glycogen.

Glycogen levels in human monocyte-derived macrophages (hMDMs) during infection by L. pneumophila were determined using the EnzyChrom glycogen assay kit (Bioassay Systems). Briefly, a total of 10 × 10^6^ PMNs were seeded into 6-well plates and infected with the wild type, ΔT*4SS*, Δ*lamA*, *lamA/C*, the catalytically inactive mutant, or formalin-killed wild type at an MOI of 1 for 1 h. As a control for glycogenolysis, PMNs were starved of glucose throughout the experiment. Cells were harvested and lysed in 25 mM sodium citrate containing 60 mM NaF (99). Following centrifugation at 13,000 × *g* for 5 min to pellet cellular debris, the supernatants were then analyzed following the manufacturers’ instructions. Degradation of intracellular glycogen was also determined using confocal microscopy. To achieve this, PMNs were plated into 24-well plates containing glass coverslips (3 × 10^6^ cells per well) and infected with either post-exponential-phase wild type or the Δ*T4SS* and Δ*lamA* mutants at an MOI of 1. At 15 min postinfection, the monolayers were fixed with 4% formaldehyde for 30 min and permeabilized with 0.1% Triton X-100 in phosphate-buffered saline (PBS) for 15 min. The monolayers were labeled with rabbit anti-*Legionella* antiserum (1:1,000 dilution); mouse anti-glycogen antibody (ESG1A9mAB, 1:50 dilution) (100), a kind gift from Ashida (Kobe University); and counterlabeled with Alexa Fluor 488 anti-rabbit IgG antibody and Alexa Fluor 594 anti-mouse IgM (1:4,000 dilution, Invitrogen) and 4′,6-diamidino-2-phenylindole (DAPI) to stain nuclei. The cells were examined by confocal microscopy using an Olympus FV1000 laser scanning confocal microscope (Olympus). Quantification of glycogen granules was performed manually by counting z-stack images (8 μM depth with 0.2 μM slices) of infected cells. Over 100 infected cells were counted for each condition and performed in triplicate.

### Determination of cytosolic glucose-6-phosphate.

To determine G6P levels in infected PMNs of 10 × 10^6^ cells were infected with either wild type, Δ*T4SS*, Δ*lamA*, the complemented *lamA/C* or formalin-killed wild-type bacteria at an MOI of 1 for 15 or 60 min. Following infection, the infected cells were collected in ice-cold PBS and rapidly homogenized on ice. Samples were centrifuged (16,000 × *g* for 20 min at 4°C), and the resulting supernatants were subjected to deproteinization using a deproteinizing sample preparation kit (BioVision) following the manufacturer’s instructions (101–103). The samples were analyzed for G6P using a high-sensitivity glucose-6-phosphate assay kit (Sigma), according to the manufacturer’s instructions, with a Synergy H1 microplate reader (Biotek).

### Determination of secreted lactate by infected PMNs.

To determine lactate secretion by infected PMNs into the culture medium, a total of 10 × 10^6^ PMNs in triplicate were infected with either wild type, Δ*T4SS*, Δ*lamA*, the complemented *lamA/C*, or formalin-killed wild-type bacteria at an MOI of 1 for 1 h. Following infection, the culture medium was retained and cooled on ice and subjected to deproteinization using a deproteinizing sample preparation kit (BioVision) following the manufacturer’s instructions (104, 105). The samples were analyzed for lactate using a lactate assay kit (Sigma) according to the manufacturer’s instructions with a Synergy H1 microplate reader (Biotek).

### Determination of bacterial killing by PMNs.

To determine killing of L. pneumophila during infection of PMNs, infections were initially monitored by confocal microscopy. To achieve this, PMNs were plated into 24-well plates containing glass coverslips (3 × 10^6^ cells per well) and infected with either post-exponential-phase wild type or the Δ*T4SS* and Δ*lamA* mutants at an MOI of 1. At 5, 15, and 60 min postinfection, the monolayers were fixed with 4% formaldehyde for 30 min and permeabilized with 0.1% Triton X-100 in PBS for 15 min. The monolayers were labeled with rabbit anti-*Legionella* antiserum (1:1,000 dilution), and intracellular bacterial morphology was analyzed by confocal microscopy. In some experiments, PMNs were coinfected with wild-type bacteria expressing mCherry with either the Δ*T4SS* or Δ*lamA* mutants. To determine if irregular bacterial morphology correlated with bacterial killing by PMNs, recoverable CFU were determined. A total of 2 × 10^6^ PMNs in triplicate were seeded into 24-well plates and infected with the wild type, Δ*T4SS*, Δ*lamA*, or the complemented *lamA/C* mutant at an MOI of 1. At 5, 15, 60, and 480 min postinfection, the culture supernatant was removed, and PMNs monolayers were lysed with sterile water. Bacterial CFU were determined by plating serial dilutions of the PMNs lysate on BCYE plates.

To determine if glycolysis contributes to the ability of PMNs to rapidly kill L. pneumophila, PMNs were pretreated with 1 mM 2-deoxyglucose prior to infection. PMNs were plated into 24-well plates containing glass coverslips (3 × 10^6^ cells per well) and infected with either post-exponential-phase wild type, Δ*T4SS*, Δ*lamA*, the complemented *lamA/C* or formalin-killed wild-type bacteria at an MOI of 1. Following 15 min infection, monolayers were fixed and processed for confocal microscopy as described above.

### Mouse infection model.

Female specific-pathogen-free, 6- to 8-week-old A/J mice were housed in specific-pathogen-free conditions within the animal care facility of the Faculty of Medicine, University of Rijeka, according to standard guidelines, and the use of animals for infection was approved by the Institutional Animal Care and Use Committee (IACUC). The incision was made through the skin of the ventral neck, the trachea was isolated, and 50 μl of the bacterial suspension in sterile saline was inoculated using a 26-gauge needle followed by 10 to 20 μl of air by intratracheal infection. A/J mice were inoculated with L. pneumophila (10^6^ CFU per mouse) wild type or Δ*lamA* mutant. The skin incision was surgically closed. At 4 and 12 h postinoculation, the mice were euthanized, and the lung was removed. The lungs were excised, minced, and incubated in RPMI 1640 medium containing 5% fetal calf serum (FCS), 1 mg of collagenase A (Sigma Chemical Company, St. Louis, MO) per ml, and 20 μl DNase per ml (Sigma Chemical Company, St. Louis, MO) for 60 min at 37°C in a shaking incubator. The cells were further disaggregated by drawing the lung homogenate repeatedly through the cell strainer prior to pelleting of the cells by centrifugation. Finally, the cells were resuspended in PBS containing 2% FCS and 1 mM EDTA. Isolation of neutrophils was performed by EasyStep mouse neutrophil enrichment kit (Stemcell Technologies, Canada), according to the manufacturer’s instructions, by immunomagnetic-negative selection. The cells were phenotyped using monoclonal antibodies specific for the following cell surface antigens: anti-CD11b (BD, Bioscience, USA) and anti-Lyg6**^+^** (eBioscience, USA). The immunofluorescence analysis was performed by BD fluorescence-activated cell sorter (FACS) Aria III flow cytometer using FACSDiva software. For determination of bacterial number within the neutrophils, the neutrophils were isolated as previously described, followed by treatment with Triton X-100. The suspension was plated on the BCYE agar and the number of bacteria determined by CFU.

### Recruitment of p47phox, lactoferrin, and elastase to the L. pneumophila phagosome.

To determine if the NADPH oxidase complex, specific, and azurophilic granules are recruited to the L. pneumophila phagosome, PMNs were plated into 24-well plates containing glass coverslips (3 × 10^6^ cells per well) and infected with either post-exponential-phase wild type, the Δ*T4SS* and Δ*lamA* mutants, or the complemented *lamA/C* mutant at an MOI of 1. Additionally, killed and opsonized S. aureus was used as a positive control for marker recruitment. At 5, 15, and 60 min postinfection, the monolayers were fixed with 4% formaldehyde for 30 min and permeabilized with 0.1% Triton X-100 in PBS for 15 min. The monolayers were labeled with rabbit anti-*Legionella* antiserum (1:1,000 dilution) in conjunction with mouse anti-p47phox (BD Transduction Laboratories), anti-lactoferrin (Abcam), or anti-elastase (Abcam) antibodies. PMNs were infected with bacteria and analyzed by confocal microscopy.

### Intracellular respiratory burst response.

A kinetic intracellular reactive oxygen species (ROS) production in neutrophils was measured as previously described (91) with modifications. Neutrophils (4 × 10^5^) were plated in 96‐well white plates, and intracellular ROS was detected by 125 μM luminol in the presence of 75 μg/ml of superoxide dismutase. Cells were stimulated with 300 nM phorbol myristate acetate (PMA) and infected with the wild type or different mutants of L. pneumophila at an MOI of 5:1 (106, 107). After the addition of phagocytic stimuli, plates were spun at room temperature at 600 × *g* for 4 min at 14°C and immediately placed in a SpectraMax L luminometer (Molecular Devices, Sunnyvale, CA, USA). Total integrated relative light units (RLUs) at 37°C were determined for 90 min using SoftMax Pro software (Molecular Devices) (108, 109). The data are represented as area under the curve from three different experiments.

The nitroblue tetrazolium (NBT) assay was also used to visualize intracellular superoxide radical production induced by the wild-type strain or different mutants of L. pneumophila. Neutrophils (1 × 10^6^ cells/ml) attached to plasma-coated glass coverslips were unstimulated or stimulated with the wild-type strain or the different mutants of L. pneumophila at an MOI of 5:1. Phagocytosis was synchronized at 600 × *g* and 14°C for 4 min, and cells were incubated at 37°C in 5% CO_2_ for 60 min in RPMI containing NBT. After incubation, cells were fixed with methanol followed by staining of L. pneumophila using antibodies. The confocal images showed reduced NBT precipitates as black deposits.
