# Supplementary figures and images for: Dot/Icm-Dependent Restriction of Legionella pneumophila within Neutrophils
Source: mBio. 2021 May 26;12(3):e01008-21. doi: 10.1128/mBio.01008-21 (PMC8262857; doi:10.1128/mBio.01008-21)

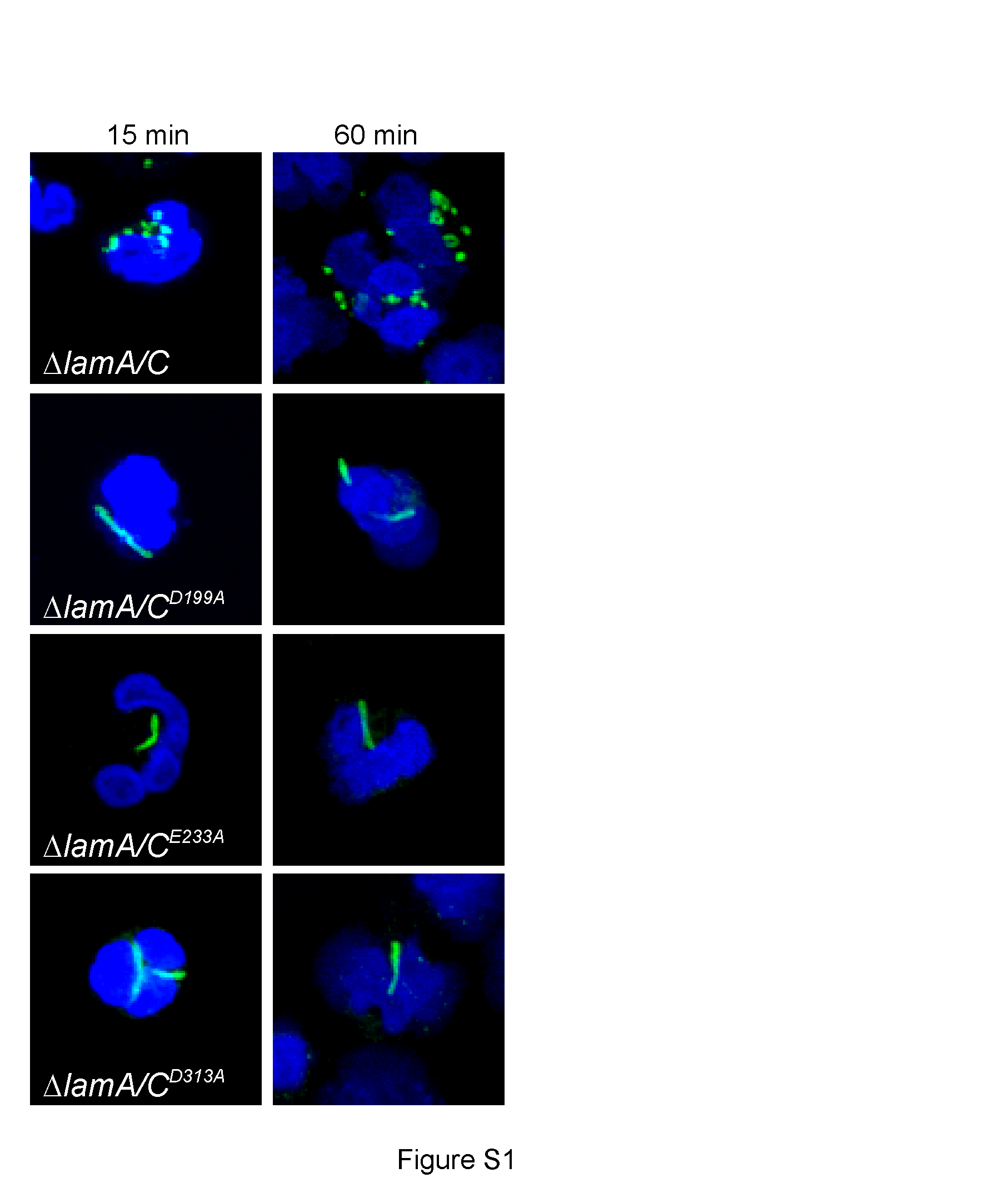

Supplement: FIG S1 [file mbio.01008-21-sf001.tif]

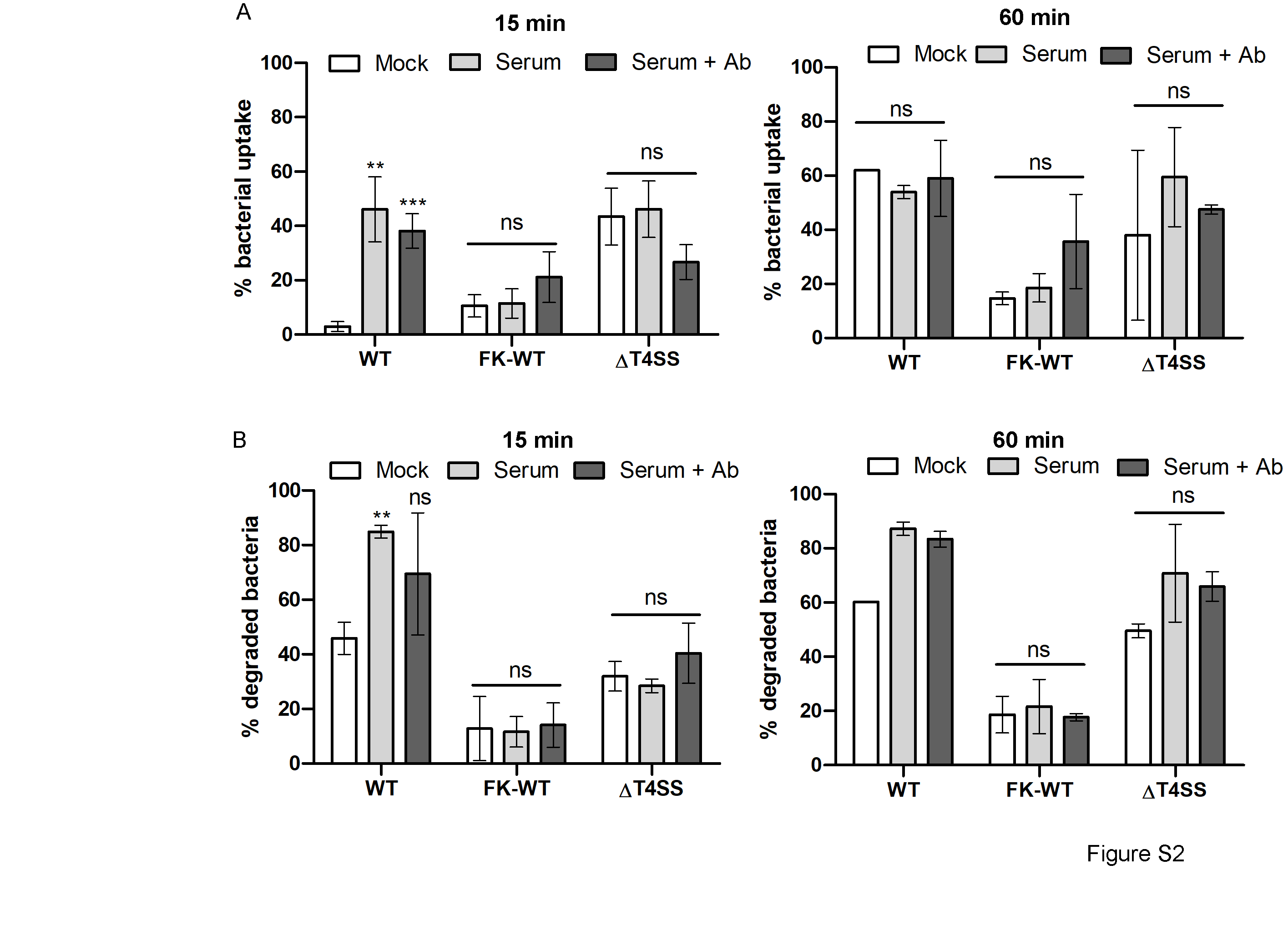

Supplement: FIG S2 [file mbio.01008-21-sf002.tif]

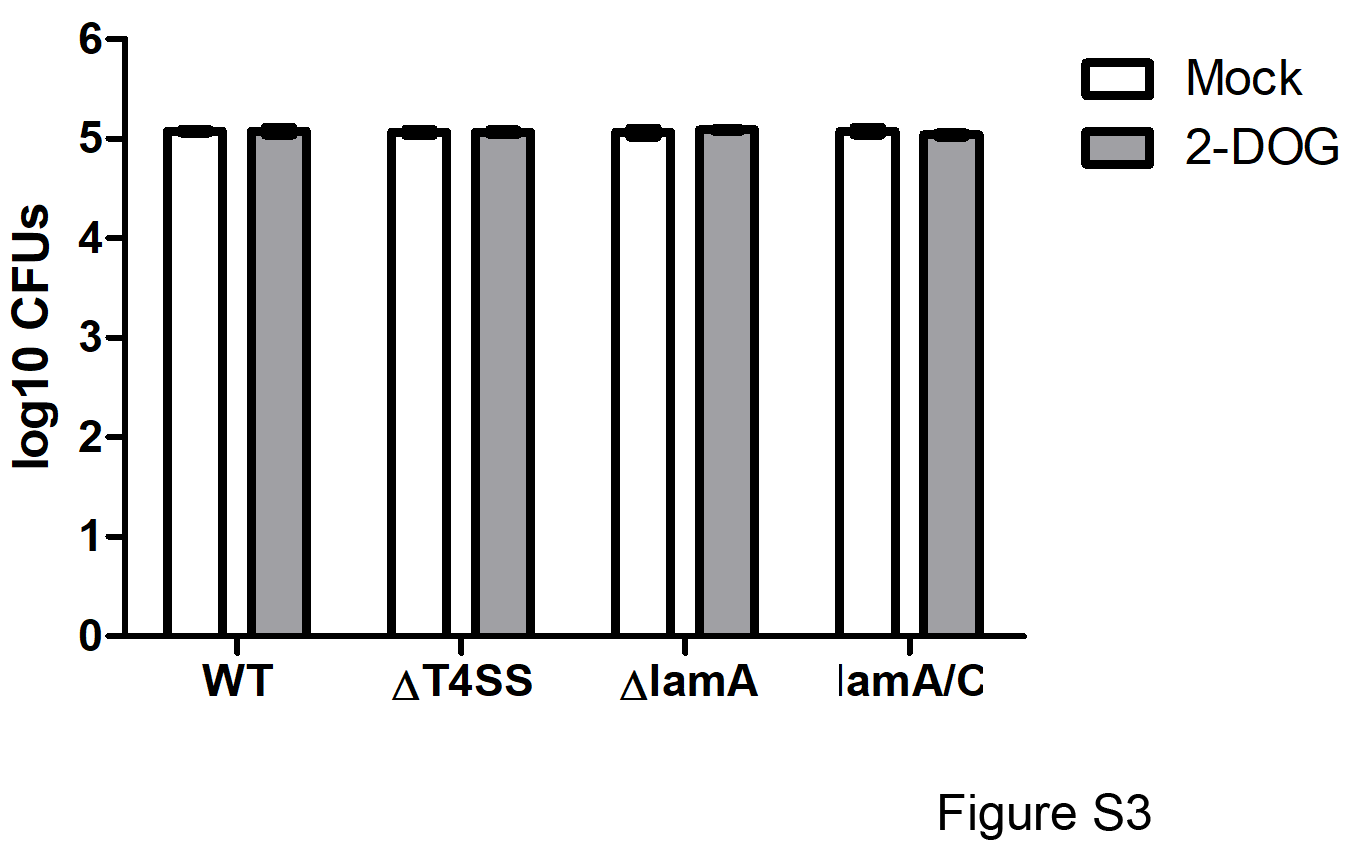

Supplement: FIG S3 [file mbio.01008-21-sf003.tif]

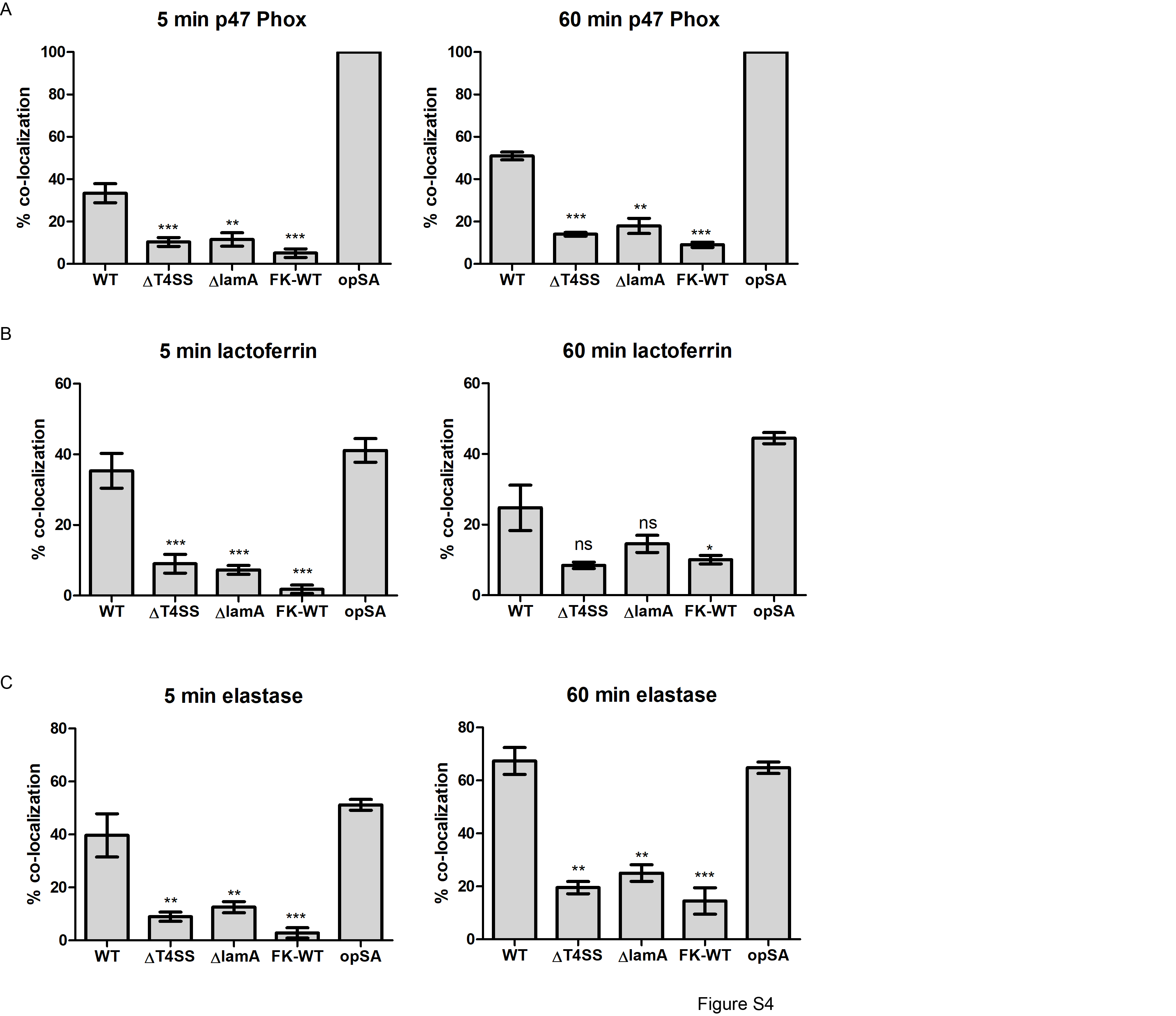

Supplement: FIG S4 [file mbio.01008-21-sf004.tif]
